# Locating unregistered and unreported data for use in a social science systematic review and meta-analysis

**DOI:** 10.1186/s13643-020-01376-9

**Published:** 2020-05-26

**Authors:** Joshua R. Polanin, Dorothy L. Espelage, Jennifer K. Grotpeter, Alberto Valido, Katherine M. Ingram, Cagil Torgal, America El Sheikh, Luz E. Robinson

**Affiliations:** 1 American Institutes for Research, 1000 Thomas Jefferson St. NW, Washington, DC 20007 USA; 2 University of North Carolina, Chapel Hill, USA; 3 Development Services Group, Inc., Bethesda, USA; 4 University of Florida, Gainesville, USA

## Abstract

Meta-analysts rely on the availability of data from previously conducted studies. That is, they rely on primary study authors to register their outcome data, either in a study’s text or on publicly available websites, and report the results of their work, either again in a study’s text or on publicly accessible data repositories. If a primary study author does not register data collection and similarly does not report the data collection results, the meta-analyst is at risk of failing to include the collected data. The purpose of this study is to attempt to locate one type of meta-analytic data: findings from studies that neither registered nor reported the collected outcome data. To do so, we conducted a large-scale search for potential studies and emailed an author query request to more than 600 primary study authors to ask if they had collected eligible outcome data. We received responses from 75 authors (12.3%), three of whom sent eligible findings. The results of our search confirmed our proof of concept (i.e., that authors collect data but fail to register or report it publicly), and the meta-analytic results indicated that excluding the identified studies would change some of our substantive conclusions. Cost analyses indicated, however, a high price to finding the missing studies. We end by reaffirming our calls for greater adoption of primary study pre-registration as well as data archiving in publicly available repositories.

The absence of evidence is not evidence of absence...

Policymakers, practitioners, and researchers rely on the results of meta-analyses to provide an overview of the findings of research conducted in a specific field and to help inform decisions about policies to implement and practices to introduce. The results from meta-analyses, however, are often at risk for bias due to, among other aspects, dissemination and publication bias. Mitigating these biases means the meta-analyst must search for and include all publicly available—and publicly *un*available—primary study results [1, 2].

Dissemination bias can lead to a meta-analyst failing to locate and synthesize all available evidence for reasons unrelated to the statistical significance of the study’s findings; for example, a study author may collect an outcome but deem the reporting of the results uninformative or of little consequence to the field. A study author may also lack the institutional support and funding to complete the project. The author’s funder may also dissuade public reporting for political or societal reasons; for example, trials funded by the US federal agencies may require specific clearance prior to release.

Publication bias, one type of dissemination bias, can lead to the meta-analyst failing to locate findings that are not published due to small or null effects. Extant evidence supports the existence of this phenomenon: nonsignificant findings are often left unpublished by study authors [3]; authors have a tendency to publish only outcomes with statistically significant effects [4]; external referees tend to provide more favorable peer reviews to studies with large or statistically significant effects [5]; favorable outcomes receive quicker time to publication [6] and receive a higher rate of citation [7]. In a review of 81 meta-analyses, encompassing 6392 primary studies, Polanin et al. [8] found that published studies’ results were 0.18 standard deviations larger than unpublished studies.

Much of the original thinking on this phenomenon coalesced in the pioneering edited book titled *Publication Bias in Meta-Analysis* [9]. The *Publication Bias* authors primarily worked from the premise that, with sufficient literature and database searching, all reported measures of a given outcome could be identified and located. The current research project is driven by a specific dissemination and publication bias question that is difficult to answer: can we locate outcomes that primary study authors never registered or reported publicly?

The purpose of this project is to answer this question, which is further divided into three sections. First, we sought to define and explicate the various types of meta-analytic data available and unavailable to synthesize. We addressed this by redefining the classic known-unknown matrix for research synthesis purposes, assigning the axes to registered-reported domains. Second, we sought to locate outcomes that primary study authors collected and possibly analyzed, but did not publish publicly (i.e., unregistered-unreported outcome data, in the newly defined matrix). We conducted a large-scale search for potentially relevant studies, emailing primary authors, and asking the authors to complete a short author query (AQ) request that identified the previously unregistered-unreported missing outcome data. The results confirmed our proof of concept (i.e., that unregistered-unreported outcome data do exist), but indicated they may prove too costly and too time-consuming to conduct in routine practice. Third, we discuss the feasibility of using the described procedures in future meta-analyses and identifying ways that primary study authors can decrease the probability of their outcome data remaining unregistered and unreported.

## Background

The former US Secretary of Defense, Mr. Donald Rumsfeld, in 2002, popularized the quotation at the beginning of this article as well as the complimentary known-unknown matrix by using the phrase “unknown-unknowns,” which refers to the phenomenon where one does not know what one does not know. The quotation garnered Mr. Rumsfeld media attention and public scrutiny, yet the “unknown-unknowns” concept was a common approach to understanding organizational or project risk management [10]. The matrix is divided into four quadrants based on the cross-section of two axes representing a continuum from known to unknown (Table 1). The upper left quadrant represents known-knowns, or recognized ideas that provide the primary basis for knowledge and decision-making. The lower left-hand corner also represents concepts that we recognize exist, but do not have information to process a decision or evaluate a risk. The upper right-hand column, on the other hand, signifies information we have available but do not understand where it might be useful, or which problem it might solve.

**Table 1:** Reconceptualizing the known-unknown matrix as types of meta-analytic outcome data

	Registered	Unregistered
**Reported**	*Registered-reported* (known-known) Found by usual systematic review processes; extracting reported summary statistics	*Unregistered-reported* (unknown-known) Found by reference harvesting; searching data repositories
**Unreported**	*Registered-unreported* (known-unknown) Found by requesting known missing data; estimating effects from other reported data; using alternative information sources	*Unregistered-unreported* (unknown-unknown) Found by “imputing” hypothetically missing data; contacting authors of potentially related studies

Terminology in parentheses was the original from known-unknown matrix; found by indicates methods to locate the four types of meta-analytic data

The remaining quadrant, the lower right-hand quadrant, has the potential to cause the most severe problems because the issue has yet to be identified. As a result, risk management experts focus on attempts to preempt risks or problems. Through the process of identification, the problem can be moved from an unknown-unknown to a known-unknown, and if a solution is identified, fully resolved in the known-known quadrant. Without thorough processing and comprehensive probing, however, the yet-to-be identified problem stays an unknown-unknown and remains solution-less.

The known-unknown matrix is a useful framework and we therefore reconceptualized it as a way of thinking about meta-analytic data. We first adopted two new axes: registration and reporting status. Registration status creates the two columns, registered and unregistered. We defined registration in the broadest possible sense, ranging from pre-registration where the authors identified outcomes intended to be measured to the “registration” of an outcome in the Methods section of a peer-reviewed manuscript. In other words, the authors articulated publicly that they intended to or had collected the outcome measure. Reporting status (reported, unreported) formed the two rows. Here we mean that the authors provided data on the outcome measure, either through summary statistics in journal article tables or full datasets in data repositories. The resulting quadrants we identified were registered-reported, registered-unreported, unregistered-reported, and unregistered-unreported.

The quadrants each represent a different type of meta-analytic data. The upper left-hand quadrant, registered-reported, is what is typically thought of as synthesized meta-analytic data. In this quadrant, the primary study authors registered their data and then published it in some form. Commonly, a primary author describes the outcome measure in a Methods section and then reports the results of the data collection in a results summary table. The meta-analyst, as a result, needs only to conduct the typical systematic review process to identify the eligible studies and synthesize the outcome data.

The lower left-hand quadrant, registered-unreported, represents what might typically be thought of as the result of potential publication bias.^1^ In this quadrant, the primary authors registered the outcome yet failed to publish the results of data collection. The meta-analyst has several options available to recover the missing outcome data. Should some information be available, it may be possible to estimate an effect size using other pieces of reported statistical information. Lipsey and Wilson [1] provided a comprehensive list of effect size transformations; Viechtbauer’s [11] powerful *metafor* R package includes the “escalc” function that provides many additional transformations. This scenario is also represented in Duval and Tweedie’s [12] “trim and fill” procedure, where the statistical algorithm identifies asymmetries in the funnel plot, presumably from missing studies or outcomes, and imputes the missing effect size. The imputed effect sizes represent the algorithm’s best guess at making an “unknown” a “known,” but the studies themselves are not identified. Instead, the effect size represents what is likely missing based on the theory of funnel plot asymmetry. Should these not be an option, the meta-analyst may query the primary author to request the missing summary statistics. Failing this request, the meta-analyst might also check other previously conducted meta-analyses or the information reported to clearinghouse (e.g., the What Works Clearinghouse’s Data from Individual Studies webpage).

Perhaps surprisingly, the upper right-hand quadrant, unregistered-reported, is a common phenomenon in meta-analyses: the study authors did not describe the measure or data collection, yet the outcome appeared in summary tables or in the final datasets published via data repositories. The former case, where data were reported in tables only, is a prime reason for conducting forward and backward reference harvesting [2]. Studies where outcomes were not articulated in the abstract or text, but only in tables, may not be identified in traditional database searches; however, other authors may have included the study in their meta-analysis. Conducting reference harvesting will increase the likelihood that the study is found. The latter case, where data are published in repositories, requires the meta-analyst to parse the documentation that accompanies the archived dataset. A helpful new search tool is the “Google Dataset Search” (https://toolbox.google.com/datasetsearch). Using this, the meta-analyst can search on key terms in the same way they would in a typical database search; any dataset matching the key terms will be identified and the meta-analyst can review it for inclusion.

The final quadrant, unregistered-unreported, is in the lower left-hand side and represents the “unknown-unknowns.” The studies and findings that derive from this quadrant represent the broader form of missingness in meta-analysis and result in dissemination bias. Data from this quandrant we do not have knowledge of and therefore we do not know are missing. It is possible that the primary author did not publish the results because some or all of the results failed to support the original hypothesis or were not statistically significant. The authors could have also considered the outcome measure of less clinical or practical importance and therefore chose not to delineate the findings. Regardless of the rationale, data from this quadrant are not known publicly and, unless the meta-analyst seeks them out, are likely to remain missing.

We identified the unregistered-unreported phenomenon as a potential problem in a recently conducted meta-analysis. While writing the meta-analysis proposal and review protocol, one of our substantive expert consultants indicated that our particular outcome of interest, cyberbullying perpetration or victimization, might have been collected in the past decade but not necessarily registered or reported early on due to its nascent cultural relevance. The expert consultant, in fact, knew of at least one case where the outcome had been collected yet not reported. We were left with a question that drives the remainder of this study: Is it possible to identify unregistered-unreported outcomes and, if so, is it possible to obtain the missing data? The following describes our efforts to collect the missing outcome information and articulates ways to prevent unregistered-unreported missing outcome data in the future.

## Methods

This work derives from an ongoing meta-analysis, which we registered on the Open Science Framework (https://osf.io/v7na6/). Along with a meta-analysis review protocol, we created a protocol for this project. An anonymized dataset, removing any identifiable information collected online or through the AQ email request described below, is published on the project’s OSF site. We also published the R scripts used to run the analyses and create the figures. The study was approved as exempt from review for being non-human subject research by the Development Services Group, Inc.’s independent Institutional Review Board (IORG0002047).

### Sample

We sought to identify primary study authors who conducted and measured an eligible outcome but did not register or report publicly the findings. To efficiently identify all potential authors, we first searched for meta-analyses that synthesized programs conducting an evaluation of school-based interventions to reduce violence, broadly. We created a search string specifically for this project: ((Aggress* OR Violen* OR bully* OR fight* OR delinquen* OR threat* OR intimidate*) AND (meta-analysis OR “meta analysis” OR “systematic review” OR “meta-analysis”) AND (intervention OR program OR policy OR practice) AND (student* OR “school-based” OR k-12 OR adolescen* OR youth OR teen OR peer*). Using the string, we searched 11 online databases using EbscoHost. We screened the resulting citations for inclusion, using the following questions: (1) Does the review include primary studies published in or after 2000? (2) Does the review include primary studies that implemented an intervention? (3) Does the review include studies that primarily used a school-based intervention? (4) Does the review include studies in which the age of the participants was P-K-12? (5) Does the review include studies that measured a school violence or related outcome? and (6) Does the review identify each included study’s citation? Any meta-analysis with an answer of “no” was excluded.

From this set of meta-analyses, we next sought to extract primary studies that might have included the unregistered-unreported outcome data. For every citation included in the meta-analysis, we screened the title and citation using the following questions: (1) Does the study’s citation indicate it was included in the meta-analysis? (2) Does the study’s title indicate an intervention or program was evaluated? and (3) Does the study’s title indicate that the content of the program was school-based and/or violence-preventing? Any study with an answer of “no” was excluded; all others were extracted to a Microsoft Excel spreadsheet.^2^

Finally, from this list of primary studies, we sought to identify the primary study’s contact author. If the email was available in the primary study, we extracted it. If not, we conducted an internet search for the first authors’ email addresses. If after sending the initial email invitation, we received a “bounceback” email indicating the address was no longer active, we sought to identify an active email address. In some cases, no active email could be located.

### Author query email request

We created an AQ email request to identify studies that collected unregistered-unreported missing outcome data. In the email sent to potential authors, we explained the purpose of the study and our interest in contacting them. We also explained the type of studies and data from those studies that were eligible, namely (1) the study included students aged 5–19 years, (2) the study implemented and evaluated a school-based intervention, and (3) the study measured cyberbullying perpetration or victimization (or a closely related measure). The email included multiple options that the authors could select depending on whether they had conducted or participated in an eligible study. If the author indicated that they had not conducted an eligible study, we recorded their participation and did not follow-up with any additional emails or questions.

If the author indicated that they had conducted an eligible study, the email link sent the author to the online request. The AQ request asked whether the author had access to the dataset. If they answered “yes,” the AQ request asked the author if they would be willing to share the summary statistics of the eligible outcomes. The authors also had the option of directly sending the full dataset, but no author chose this option. If the author answered “no” to having access to the dataset, then the AQ request asked if they had access to the summary statistics of the eligible outcomes. If at any point the authors indicated they were willing to share summary statistics, then a form was populated where authors could input the means, standard deviations, and sample sizes for each condition for each outcome. If the authors answered “no” to every question, there was one final option at the end to send a PDF or citation of a study where the eligible outcomes might be located. Finally, we also stated in the email invitation that authors could send us study information, citations, alternative authors to contact, or PDFs of potentially eligible studies and outcomes. In these cases, the author did not complete the online request but their response was recorded.

### Procedure

The 2nd author’s laboratory email account was used to send the AQ email request to potential authors. Each author was sent an anonymous link to the request, developed in Qualtrics. Authors received two follow-up emails if they did not respond in any manner to the original request. Each response was categorized (e.g., out of office, bounce back, personal reply).

### Analysis

We conducted several descriptive and exploratory analyses. Following the meta-analytic processes specified in our review protocol, we estimated standardized mean differences corrected for small-sample bias (Hedges’ *g* [13];) from all eligible outcomes. We extracted study characteristics and effect size data from the newly included studies received from the AQ email request, adding the studies to the results of the ongoing meta-analysis. We then estimated meta-analytic models, comparing the results with and without the newly added studies, using a random-effects model accounting for effect size dependency using robust variance estimation [14]. After exploring differences in effect size heterogeneity, we conducted exploratory analyses comparing the results of moderator analyses, where one set of analyses included—and one set excluded—the newly added studies. We chose three moderators, all of which we considered exploratory because we did not pre-register the analyses. We selected the moderators because they illustrated commonly tested moderators of interest. Finally, as a way of estimating future costs, we asked project staff to closely record their time for this activity on the project. From these estimates, we calculated the cost to the project as well as a per-study-added cost. All analyses were conducted in R [15] the meta-analyses were conducted using *robumeta* [16], and the graph was created with *ggplot2* [17].

## Results

For the search of studies from meta-analyses, we searched 11 traditional online databases and found 511 potential meta-analyses. From the 511, the abstract screening process resulted in 78 potentially eligible meta-analyses. After retrieval and full-text screening, 57 meta-analyses were deemed eligible. From the 57 meta-analyses, we extracted 894 primary study citations. The 894 study citations resulted in 686 unique individuals. We located 609 working emails, either from the published article, from the article’s supplemental materials, or through an Internet search. It is notable that more than 1 in 10 primary authors’ emails were not retrievable, which highlights the concerning issue of contacting corresponding authors for meta-analysis generally.

**Fig. 1:**
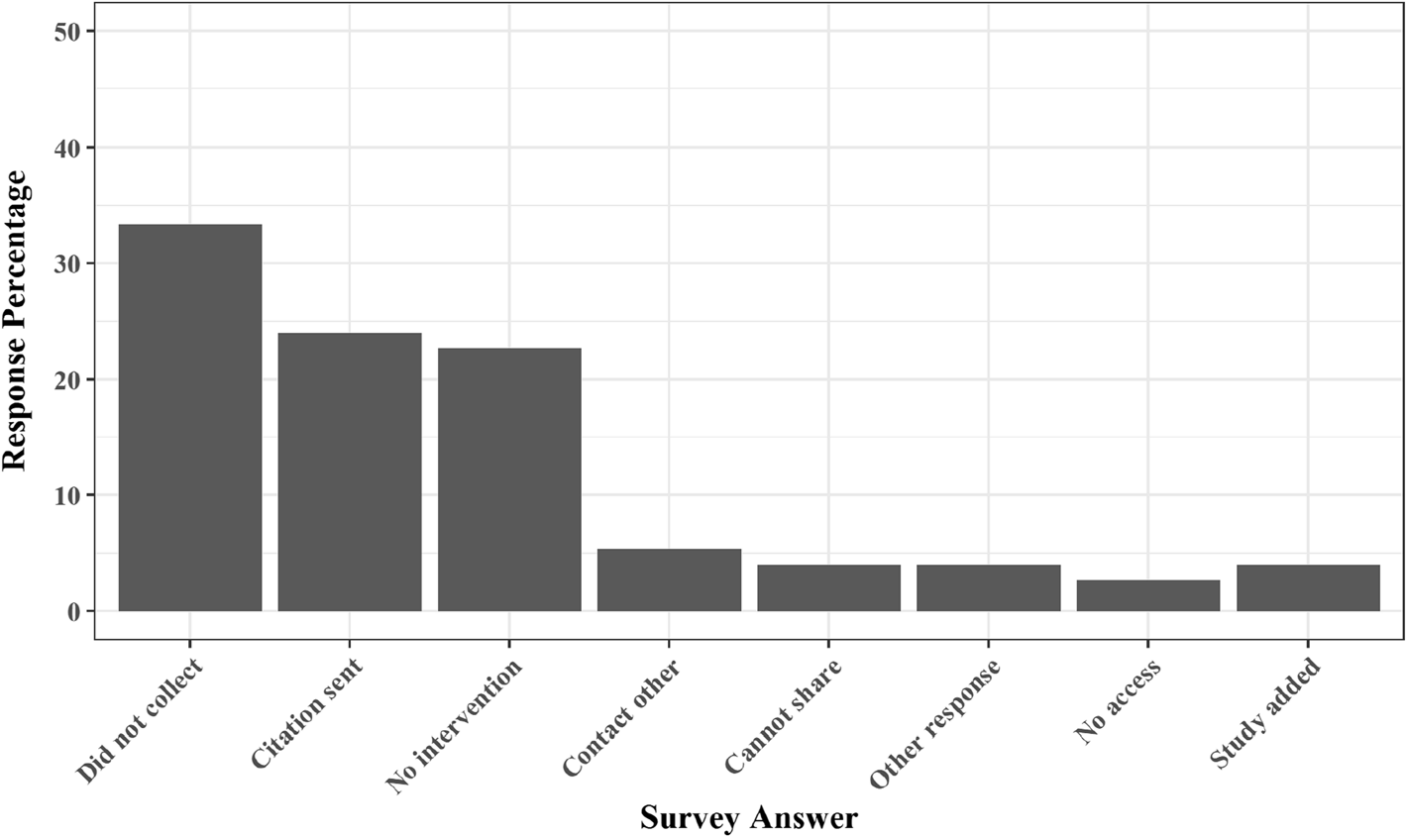
Responses from authors. *N* = 75

We sent emails asking for participation in the project, and the location of the unregistered-unreported outcome data, to the 609 unique email addresses. Figure 1 illustrates the responses (or non-responses) received. Almost 7 in 10 authors did not reply in any manner (*n* = 432, 70.9%), which could have meant that they did not collect the requested information, they were not willing to share the missing information, or they were not willing to respond to the request. In any case, this large proportion of non-response was not surprising given the typical response rates requesting outcome data. Additional non-responses resulted from out of office replies (*n* = 25, 4.1%) and nonfunctional email addresses (*n* = 77, 12.6%). In both cases, we attempted to follow-up by searching the Internet for an additional email address or sending additional email requests, respectively. These attempts received no responses. In total, 75 authors (12.3%) responded, either completing the AQ request via the anonymized link (*n* = 29, 4.8%) or replying to the email directly providing the requested information (*n* = 46, 7.6%).

The responding authors provided a variety of information (Fig. 1). A plurality of respondents indicated that they did not collect the requested information (*n* = 25, 33.3%). Many others responded by sending us a study that they considered potentially eligible but upon further review were not (*n* = 18, 24%). The third largest proportion of responses derived from individuals who did not conduct an eligible study (*n* = 17, 22.7%). Smaller categories of responses included individuals who asked us to follow-up with someone else, each of whom did not respond or send us data (*n* = 4, 5.3%); individuals who would not share their data because of IRB concerns (*n* = 3, 4.0%); individuals who said they would get back to us but did not (*n* = 3, 4.0%); and individuals who said they conducted an eligible study with an eligible outcome but no longer had access to the dataset (*n* = 2, 2.7%).

Most importantly, three individuals, representing three papers, responded by sending an eligible study and outcome data. The first eligible response [18] provided summary statistics for one truly unregistered-unreported missing outcome. The study’s results indicated a positive intervention effect. The second eligible response [19] sent a published manuscript from a large network of evaluation studies. The missing outcome data would have been difficult to locate because it was not discussed in the abstract and only reported within a table. The study contributed three effect sizes, two were not statistically significant. Finally, the third eligible response [20] provided a yet-to-be-published study. The study contributed two effect sizes, both statistically significant intervention effects. The inclusion of the six effect sizes increased the overall average treatment effect from − 0.14 to − 0.17, but the 95% confidence intervals almost completely overlap (without new studies − 0.21, − 0.06; with new studies − 0.24, − 0.09) and a *t* test of difference between the original and newly added studies was not statistically significant (*t*(2) = 2.26, *p* = 0.14).

The overall meta-analytic average represents an important reason to seek out and include the missing studies, regardless of the study’s inclusion on the overall meta-analytic average. However, the inclusion of additional eligible findings can impact effect size heterogeneity as well as the results of moderator analyses. To illustrate this point, we estimated the heterogeneity statistics for each dataset (without and with the new studies) and then selected and tested three potential moderators on the two datasets. Note that all three moderator analyses were exploratory, and readers should not interpret these results as substantial; rather, we present these results to further elucidate the proof of concept. Table 2 delineates the findings.

**Table 2:** Exploratory moderator analyses: adding studies added from author query

Includes added studies?	Level	*k* (ES)	Meta-analytic average (SE)	95% CI	*t* value (*p* value)
No					2.36* (.05)
	Local	40 (121)	− .16 (.04)	− .24, − .08	
	Non-Local	7 (41)	− .04 (.03)	− .12, .05	
Yes					0.83 (.42)
	Local	41 (122)	− .18 (.04)	− .27, − .09	
	Non-Local	9 (49)	− .12 (.05)	− .26, .02	
No					1.06 (.30)
	US	31 (110)	− .17 (.04)	− .25, − .09	
	Non-US	16 (55)	− .09 (.07)	− .24, .08	
Yes					0.62 (.54)
	US	32 (113)	− .19 (.04)	− .27, − .10	
	Non-US	18 (58)	− .13 (.08)	− .29, .03	
No					0.82 (.51)
	Universal	43 (156)	− .12 (.03)	− .19, − .05	
	Tertiary	4 (9)	− .42 (.36)	− 2.48, 1.65	
Yes					1.59 (.20)
	Universal	45 (161)	− .13 (.04)	− .20, − .06	
	Tertiary	5 (10)	− .60 (.29)	− 1.57, .36	

“Includes added studies” column indicates if the results include the additional 3 studies and 6 effect sizes; local: school, school district, or city; non-local: region, state, or nation; *k* number of studies, *ES* number of effect sizes, SE standard error, *CI* confidence interval. *t* value and *p* value represent the test statistics from the meta-analytic moderator analyses

The results revealed several findings of interest. We found important differences in the estimates of effect size heterogeneity: the heterogeneity without the new studies (*τ*^2^ = .027) was 57% smaller compared to the heterogeneity with the new studies (*τ*^2^ = .063). We also found differences among the moderator analyses; specifically, of the three moderator analyses, the locality of the intervention resulted in substantively and statistically different conclusions. Without the three additional studies, the results indicated that local sampling designs—where a single school, district, or city was the focus of the intervention—had a statistically significantly larger intervention effect, relative to non-local sampling designs (e.g., state, region, or national samples). After including the newly added studies, however, there was no longer a statistically significant difference between the two sampling designs. Although the results of the other two moderators showed no statistically significant differences, the locality moderator analysis illustrated the potential consequences of unregistered-unreported meta-analytic data.

A last consideration for this project was the cost of the process to find unregistered-unreported data. Ten individuals worked on some element of the project, including leadership (*n* = 3), research staff at a social science research firm (*n* = 3), and graduate or undergraduate students (*n* = 4). The leadership team estimated that their contribution totaled approximately 14 h, the research staff amounted to 70 h, and the students totaled 63 h. In addition, the team met numerous times as part of the usual weekly project meetings; this portion of the meeting typically lasted between 10–20 min and the team met and discussed this project approximately 15 times for an additional 4 h. Therefore, across all staff, the project totaled approximately 176 h or 58.7 h per study. Assuming an approximate $40/h per person cost, the inclusion of each study cost $2348 or $7044 total US dollars to include all three studies.

## Discussion

### Summary

We posited that meta-analytic data may be categorized into four distinct types. We defined two axes, registered and reported, as a way to conceptualize each: registered-reported, registered-unreported, unregistered-reported, and unregistered-unreported (Table 1). Three of the four types have established systematic review methods used to identify and include the meta-analytic data. The fourth type, unregistered-unreported, remains a risk to the validity of meta-analyses, and few systematic efforts may be undertaken to collect the missing outcome data.

Our meta-analysis project sought to identify the unregistered-unreported missing outcome data. We conducted a wide-ranging search for potentially related meta-analyses, identified primary studies within each that may have collected the missing outcome data, found the emails associated with the primary study authors, and sent a request for the missing outcome data. We identified over 600 unique authors and sent emails to each. Many authors failed to respond (70%). Of the authors that responded, most stated that they did not conduct an eligible study, did not collect an eligible outcome, no longer had access to the data, had IRB concerns, or failed to follow-up after numerous attempts. Despite the poor response rate, we confirmed the proof of concept by collecting three studies that would likely not have been included otherwise. Although the overall meta-analytic effect size differed little, the heterogeneity of effects differed considerably and one of the three moderator analyses we conducted would have resulted in a different finding. We estimated that procuring these three studies required about 176 additional hours of labor.

### Feasibility for meta-analysis

We initially posited that a meta-analytic reviewer must assume some level of risk due to dissemination and publication biases. Thus, reviewers must continue to assume this risk until methods that can provide some level of certainty that all research conducted is available to synthesize are popularized. We discuss below what primary study authors, policymakers, program officers, and editors, all of whom maintain the systems for primary study conduct and publication, must do to work toward this goal. Until such a time, we are left to consider: what can the meta-analyst do to reduce the risk of dissemination and publication biases? In other words, should we expect that reviewers go to the lengths described in our study to find all completed research?

The question is difficult to answer because few meta-analysts attempt to collect unregistered-unreported data and none, to our knowledge, has considered such a large-scale attempt at AQ email requests. In other fields, medical or environmental sciences for example, it may be easy to locate potentially eligible studies based on their context. Perhaps a reviewer can easily locate studies where, if one outcome was registered and reported, it is almost certainly the case that a related measure was registered, whether or not it was reported. The search for adverse effects is one such example [21]. For these fields or areas of research, the cost of using our research design may therefore be considerably lower and meta-analysis methodologists should consider asking future reviewers to do the same.

A related question is whether any costs, large or small, are worth spending at all. The results of our comprehensive AQ email request resulted in a gain of three studies. All three studies would likely *not* have been included in our completed review. Of note, these three studies represent a contribution of 5.4% new studies to a database that originally included 56 studies. While we regarded any increase in the number of studies included is a success, some may argue that adding such a small number of new studies is not worth the cost. Our counterargument is this: what increase in the percentage of studies in a review would be high enough to justify using our procedures? Any proportion posited will be based on intuition rather than empirical estimation. Until such a time when we have all available data and/or datasets publicly available, we argue that if a reviewer has the resources, using our procedures ensures that most information is found and included in a review.

Finally, we recognize that our review may be unique in that it received external funding. Many high-quality reviews lack sufficient funding, or any funding at all, and therefore will not have the resources to use our procedures. We do not view this problem as a fatal flaw, rather a limitation and caution that the reviewer must acknowledge when summarizing the results. No review will find *every* piece of data ever collected on a topic, but some reviews come closer than others. Meta-analysts must be willing to acknowledge when their own reviews fall short relative to others.

### Reducing the probability of unregistered-unreported outcome data

While previously conducted primary studies, and the meta-analyses that synthesize their findings, may incur unregistered-unreported problems, future primary studies need not. We see at least three ways that primary authors, and those that help to produce primary research, can help to reduce the problem: pre-registration of outcome data, clear and comprehensive of outcome reporting, and complete data archiving.

We join the call to encourage primary study authors to pre-register their studies [22]. Doing so clearly promotes studies from the left-hand columns to the right-hand columns. It does not prevent a known-unknown problem, traditionally known as publication bias. However, the pre-registration of outcome data allows meta-analysts to find and synthesize the collected data. We are particularly encouraged by the avenues available to primary authors for pre-registration: Open Science Framework (OSF), Registry of Efficacy and Effectiveness Studies (REES), ClinicalTrials.gov, Evidence in Governance and Politics (EGAP), American Economic Association’s Registry for RCTs, or the Registry for International Development Impact Evaluations. Primary study funders must also establish incentives for primary study authors to pre-register funded studies. Meta-analysts may then comprehensively search these registries and decrease the risk of not including collected outcome data.

Equally important, primary study authors should continue to heed calls to report and publish their results, regardless of statistical significance or confirmation of hypotheses. The funders that support researchers’ work, as well as the journal editors who gatekeep the flow of information, must also be willing to allow the publication of less-than-flattering or confirmatory findings. We recognize publishing unfavorable findings has the potential to lead to short-term funding and prioritization consequences, but the long-term gains for science and the science of evidence synthesis should, in our opinion, outweigh these consequences.

In addition to registration and reporting of outcome data, we call for the continued accumulation of knowledge in data repositories. Funders of research studies, as well as primary study authors, have an obligation to distribute their data publicly. We call for this action as meta-analysts who seek to reduce unregistered-unreported outcome data, but also for future researchers who may not seek to synthesize individual studies and instead conduct new or updated primary analyses. Of course, the reporting of outcome data also ensures that meta-analysts can synthesize it, thus reducing any risk from publication bias.

### Limitations

Several limitations should be noted. The biggest limitation is that our method to locate unregistered-unreported meta-analytic data, while rigorous and large in scale, may not in fact have netted all conducted research findings. The results from these missing trials are subject to the same dissemination and publication biases that other meta-analyses suffer. We have no definitive way of knowing truly how many unregistered-unreported studies or findings from studies exist. Relatedly, our response rate was encouraging yet still disappointingly lower than expected. We hypothesize that at least one to two authors who failed to respond possess unregistered-unreported eligible outcome data.

## Conclusions

We began this study because we were concerned about unregistered and unreported outcome data on a relatively new construct. We did not originally conceptualize the potentially missing outcome data in this manner, but the results of this study lead us to posit this new frame of reference. The results of this study confirmed our proof of concept as one that continues to pervade the field of systematic reviews and meta-analyses. Meta-analysts must do all possible, within their financial and other resource constraints, to seek out all types of meta-analytic data, not only those unregistered and unreported. Primary study authors must continue to increase what is known about the studies they have conducted. With all parties working together, the risks associated with publication biases can decrease and the validity of meta-analytic research can continue to increase.

## Data Availability

The datasets generated during and/or analyzed during the current study are available in the Open Science Framework repository (https://osf.io/v7na6/).

## Footnotes

1 Note that the lower left-hand quadrant might also be considered the result of dissemination bias, which, as we describe, encompasses publication bias.

2 Note that because we screened citations, and not abstracts, our results included more false positives (i.e., we were more lenient and included some studies that may not have been evaluations of interventions). Some participants responded to our AQ email request simply to tell us that they had not conducted an eligible evaluation.
